# Serological Patterns of Brucellosis, Leptospirosis and Q Fever in *Bos indicus* Cattle in Cameroon

**DOI:** 10.1371/journal.pone.0008623

**Published:** 2010-01-21

**Authors:** Francesca Scolamacchia, Ian G. Handel, Eric M. Fèvre, Kenton L. Morgan, Vincent N. Tanya, Barend M. de C. Bronsvoort

**Affiliations:** 1 The Roslin Institute at the Royal (Dick) School of Veterinary Studies, University of Edinburgh, Roslin Biocentre, Roslin, Midlothian, United Kingdom; 2 Institute of Immunology and Infection Research, School of Biological Sciences, University of Edinburgh, Edinburgh, United Kingdom; 3 Department of Veterinary Clinical Sciences, University of Liverpool, Leahurst Veterinary Teaching Hospital, Neston, Wirral, United Kingdom; 4 Ministry of Scientific Research and Innovation, Yaounde, Cameroon; St. Petersburg Pasteur Institute, Russian Federation

## Abstract

Brucellosis, leptospirosis and Q fever are important infections of livestock causing a range of clinical conditions including abortions and reduced fertility. In addition, they are all important zoonotic infections infecting those who work with livestock and those who consume livestock related products such as milk, producing non-specific symptoms including fever, that are often misdiagnosed and that can lead to severe chronic disease. This study used banked sera from the Adamawa Region of Cameroon to investigate the seroprevalences and distributions of seropositive animals and herds. A classical statistical and a multi-level prevalence modelling approach were compared. The unbiased estimates were 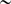
20% of herds were seropositive for *Brucella spp.* compared to 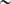
95% for *Leptospira spp.* and 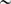
68% for Q fever. The within-herd seroprevalences were 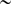
16%, 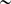
35% and 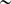
39% respectively. There was statistical evidence of clustering of seropositive brucellosis and Q fever herds. The modelling approach has the major advantage that estimates of seroprevalence can be adjusted for the sensitivity and specificity of the diagnostic test used and the multi-level structure of the sampling. The study found a low seroprevalence of brucellosis in the Adamawa Region compared to a high proportion of leptospirosis and Q fever seropositive herds. This represents a high risk to the human population as well as potentially having a major impact on animal health and productivity in the region.

## Introduction

Zoonoses or diseases transmitted from animals to man, have been recognised as important public health issues for centuries and much of the early history of veterinary science was focused on the control of diseases such as bovine tuberculosis. Ungulates, in particular, are known to carry at least 315 zoonotic pathogens [1] and many emerging and re-emerging infectious disease problems globally are zoonotic [2]. In spite of the clear need to understand these diseases in the animal populations where they may be maintained [3] the veterinary and medical professions need to work closely on infectious disease research in multidisiplinary teams to be successful in tackling many of these diseases. There is a clear and urgent need for this in sub-Saharan Africa (SSA) where the public health and veterinary infra-structures have virtually collapsed through neglect and enforced privatisation.

Brucellosis, caused by bacteria of the genus *Brucella*, is a significant worldwide infectious disease of domesticated animals and wildlife. In animals it is characterized by reproductive failure in females and sterility in males. In man it causes a range of symptoms but typically an undulating fever and is one of the most ancient described zoonosis [4], [5]. *B. abortus* is the cattle adapted species and typically is a major abortive agent. It has been the object of successful eradication campaigns in many countries in the developed world. *B. melitensis* may also cause abortion in cattle, although it is mainly associated with sheep, goats and wildlife [6]. Brucellosis is widespread with varying prevalences across Africa, with some areas reportedly having up to 30% seroprevalence. The state of knowledge was recently reviewed by McDermott and Arimi [7], who highlighted its relative importance in cattle, sheep, goats, pigs and wildlife across the main livestock production systems in SSA.

Leptospirosis is a zoonosis of ubiquitous distribution, caused by infection with pathogenic spirochetes belonging to the genus *Leptospira*. They infect a wide spectrum of hosts, including mammals, reptiles, birds and amphibians. They pose a significant public health problem of increasing concern as well as great impact on the reproductive efficiency of livestock [8]–[11]. Cattle are the maintenance host for *Leptospira borgpetersenii* serovar Hardjo (subtype hardjobovis) and *Leptospira interrogans* serovar Hardjo (subtype hardjoprajitno), which are serologically indistinguishable but genetically distinct [10]. A variety of clinical illnesses are seen when a cow becomes infected for the first time: abortion, mastitis, loss of milk and calves may be stillborn, weak or clinically normal but infected. Infertility associated with persistent infection is the most important economic consequence. Infection is usually transmitted directly by contact with infected urine, run-off water or abortion fluids from infected animals. The situation regarding leptospirosis in Africa is mostly unknown and rarely documented outside South Africa [12], although it is associated with high rainfall regions in cattle in South Africa. Symptoms of leptospirosis in man include high fever, severe headache, chills, muscle aches, and vomiting, and may include jaundice, red eyes, abdominal pain, diarrhea, and/or a rash. The symptoms in humans appear after a 414 day incubation period following contact with infected urine from animals.

Q fever is a highly contagious zoonotic disease caused by the intracellular pathogen *Coxiella burnetii*. Multiple hosts can serve as a reservoir of infection, but aborting domestic ruminants are typically the main source of the bacterium in humans and animals. The disease has been recognised since the 1930s and has a worldwide distribution with the exception of Antarctica and New Zealand [13], [14]. All domesticated ruminants are susceptible but, with the exception of reproductive failures such as abortions, stillbirths, infertility and weak offspring, animals are usually asymptomatic and can remain chronically infected [15]–[17]. Infection in man results from inhalation of airborne contaminated particles and from contact with the milk, urine, faeces, vaginal mucus, or semen of infected animals. The most common manifestation in man is a flu-like illness which can progress to an atypical pneumonia, which can result in a life threatening acute respiratory distress syndrome. The chronic form of Q fever is virtually identical to endocarditis which can occur months or decades following the infection. It can be considered the most infectious disease in the world, as a single bacterium is sufficient to cause infection.

This paper presents a serological analysis of exposure to *Brucella spp.*, *Leptospira spp.* and *Coxiella burnetti* in cattle in the Adamawa Region of Cameroon in 2000. The presence of antibodies and hence exposure to these pathogens was measured using ELISAs. The study used banked sera from a previous population-based survey of foot-and-mouth disease in the region. We have used both a conventional estimation approach and a Bayesian framework for the analysis. One of the major problems of surveys and surveillance data is that the results are generally based on an indirect measure of disease or exposure such as a serological test. Few studies appear to include any adjustment for the imperfections or uncertainties in the testing systems they use and therefore risk giving both a biased estimate of seroprevalence and a higher degree of confidence than is actually supported by the data. This may be partly because there is a shortage of reliable test parameter estimates in the literature for well defined populations and also because test parameters are populations specific and the performance of many diagnostic tests in tropical settings is known to be lower [18]. Our approach has been to incorporate prior knowledge about the test parameters where available and use these to estimate the true seroprevalence adjusting for both diagnostic test performance and the study design. These diseases are important both because of the direct impact on livestock production but also because of the potential impacts on human health. Understanding the patterns of these diseases in the livestock populations is critical for both the veterinary and public health services if sensible priorities are to be set and controls are to be implemented.

## Materials and Methods

### Samples

**Figure 1:**
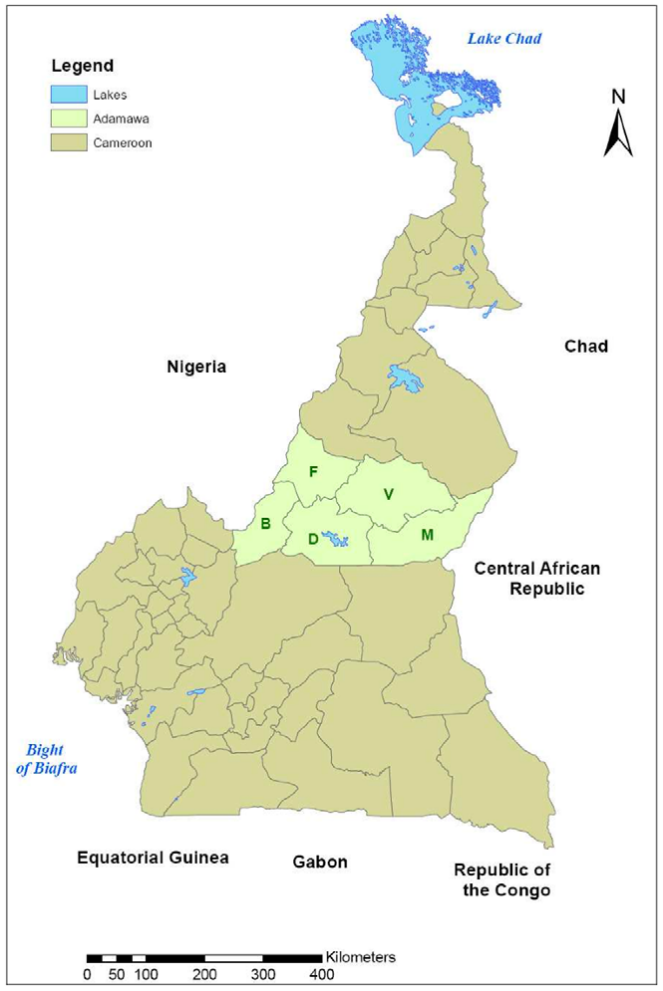
Political map of Cameroon showing the Adamawa Region and the five administrative Regions within it. (V = Vina; M = Mbere; D = Djerem; B = Mayo Banyo; F = Faro et Deo).

The samples used for this investigation were originally collected as part of a study of foot-and-mouth disease in Cameroon. The study population has been described in detail [19]. Briefly, the study area was the Adamawa Region of Cameroon, an area of approximately 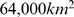 lying between latitudes 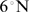 and 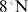. It is the main cattle producing region of Cameroon and is divided into five administrative divisions (Vina, Mbere, Mayo Banyo, Djerem and Faro et Deo), with 88 Ministry of Livestock, Fisheries and Animal Industries (MINEPIA) veterinary centres distributed across it (Figure 1). A database of 13,006 herds constructed from rinderpest vaccination records was used as the sampling frame. A cross sectional study design was used and a stratified, two stage random cluster sample of cattle herds was selected. Sample size was calculated on the basis of an assumed FMD herd seroprevalence of 50% [19].

Herds were visited between April and October 2000. Samples were collected from 146 herds. Five adult (more than 24 months of age) and five juvenile (8 to 24 months of age) samples were collected from the majority of the herds, producing 1377 individual samples in total. Blood was sampled by jugular venepuncture and allowed to clot. At the end of each day the blood samples were centrifuged in the field and approximately 3.5ml of serum was separated from each and divided into two 1.8ml cryovials (Nunc, Thermo Fisher Scientific). The samples were kept at 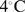 in a portable gas refrigerator until they could be frozen and stored at 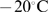, then transported to the UK on dry ice. They have since been stored at the FMD World Reference Laboratory (WRL), Pirbright, at 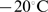.

### Diagnositic Tests

#### Brucella cELISA

The cELISA Brucella diagnostic kit is based on detection of the lipopolysaccharide (LPS) antigen of smooth *Brucella* strains. The immunodominant epitope of the LPS is the O-chain which is a homopolymer of 1,2-linked N-acylated 4-amino-4, 6-dideoxy-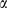-D-mannopyranosyl residues [20]. The cELISA was provided and performed by VLA staff according to the O.I.E. Manual of Standards for Diagnostic Tests and Vaccines using the 16M Melitensis strain as antigen and OPD as the chromogen, stopped with Citric acid. The optical density (OD) was read at 450nm and the percentage OD of the conjugate (% OD) were calculated as the average OD of the paired sample wells divided by the average OD of the four conjugate wells on the plate. The cELISA used a monoclonal antibody specific to the O-chain polysaccharide portion of the *Brucella* LPS [21]. The standard %OD cut-off of 70% was used initially for interpretation of results but 60% and 50% cut-offs were also explored in the latent class analysis. Using the recommended cut-off and based on the literature, the prior estimates for Se and Sp were 97.8% and 98.6% respectively. All test results were read blind and all results used in this analysis were from the first test unless a plate failed in which case the whole plate was repeated to ensure the controls were within the validation limits.

#### *Leptospira hardjo* ELISA

The Linnodee Lepto Kit (Linnodee Animal Care, Ballyclare, UK) was used to screen the cattle sera for antibodies to *Leptospira hardjo*. This is a monoclonal antibody capture ELISA kit that detects an antibody response to a LPS outer envelope epitope common to both *Leptospira borgpetersenii* serovar *Hardjo bovis* and *Leptospira interrogans* serovar *Hardjo prajitno* [22]. Sera were diluted 1∶50 in the kit dilutent and 100
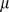
l was added to a well. Positive and negative controls were run in triplicate on each plate. The plates were incubated at 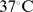 for 40 minutes with gentle shaking, then washed with buffer 4 times. 100
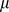
l of conjugate was added and the plates covered and incubated at 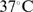 for a further 30 minutes with gentle shaking, then washed 4 times with the supplied buffer. Finally 100
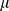
l of substrate was added to each well and the plate incubated in the dark at room temperature for 12 minutes. 50
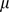
l of stop solution was added and the plates read at 450nm. The test results were expressed as a ratio of the test sample and a mean positive control serum. A sample was recorded as positive if the ratio was greater than the negative cut-off, where the latter was calculated using sera controls using the formula:
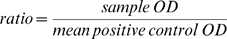
(1)

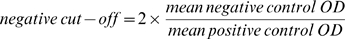
(2)


Using the recommended cut-off and based on the literature the prior estimates for Se and Sp were 82.8% and 96.5% respectively. The small sample sizes these are based on is reflected in the higher uncertainty in the priors (Table 1).

**Table 1:** Priors used for each diagnostic test for modeling true seroprevalence.

Parameter	Brucella	Leptospia	Q fever
seA	3428	44	17
seB	77	9	1
spA	7860	217	22
spB	111	8	1

#### Q fever ELISA

A commercial ELISA kit (Chekit-Q-fever, Bommeli, IDEXX Laboratories, Broomfield, CO) was used to screen each serum sample for IgG antibodies to *Coxiella burnetii* based on *C. burnetii* phase I and II purified antigens, where 100
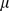
l of 1∶400 dilutions of sera were added to the plate with pre-coated *Coxiella burnetii* antigen and incubated for 60 minutes at 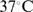. After incubation the plates were washed 3 times and 100
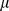
l of anti-ruminate IgG conjugate added and incubated for a further 60 minutes. The plates were washed 3 times and 100
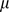
l of TMB substrate added to each well and left at room temperature for 15 minutes. The reaction was stopped using the stop solution provided and the plates read at 450nm. Plates where the positive control OD exceeded 2.0 or the negative control OD exceed 0.5 or if the difference between the controls was 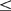
0.3 were rejected and rerun. Samples were run as single spots and 2 positive and 2 negative controls were included on each plate. The % value was calculated using the following formula expressing the OD of the sample as a percentage of the positive controls adjusted for the background OD:
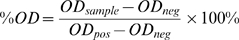
(3)


As recommended by the manufacturer, animals were considered to be positive if they had an optical density percentage (%OD) 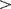
40, negative if OD%
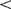
30 and ambiguous if between 30 and 40%. Using the recommended cut-off of 40% and based on the literature the prior estimates for Se and Sp were 94.5% and 95.5% respectively. The small samples these are based on is reflected in the higher uncertainty in the priors (Table 1).

### Statistical Analysis

The apparent/test based seroprevalence estimates were calculated using the *svy* command in Stata 9.0 (Stata Corporation, Texas, USA). The animal-level region-wide seroprevalence variance estimates (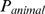), were adjusted using herd as the clustering variable and Division the stratification variable. For the estimates of the proportion of seropostive herds (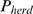), the data set was collapsed to the herd-level and each herd classed as seropositive if one or more animals were test positive for the initial analysis and two or more for the adjusted analysis. Both the 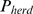 and 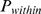 variance estimates included adjustment for the study design with veterinary centre as the primary and herd the secondary sampling units, Division as the stratification variable and a weighting to adjust for missing herds from the original sample [19], [23]. All confidence intervals are given as 95% intervals for ease of comparison between estimates. None of these estimates include an adjustment for the test sensitivity or specificity.

### Modelling

A prevalence model was developed based on the framework used by Branscum *et al.* [24]. Counts of test positive animals in each herd were assumed to be distributed:
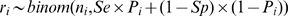
(4)where 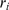 is the count of test positive animals in herd 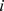, 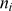 is the number of animals sampled in herd 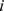, 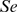 and 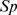 are the test sensitivity and specificity and 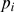 is the prevalence of sero-conversion in herd 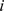. The within herd prevalence, 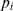 is assumed to be distributed as a mixture:
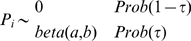
(5)


In the absence of other data the probability that a herd was sero-positive (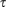) was given a vague prior distribution 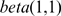. The within herd prevalence used the parameterisation from Branscum *et al.* [24] permitting it to be specified with hyper parameters describing the uncertainty of the mean within herd seroprevalence and a term related to its variance.
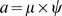
(6)

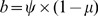
(7)


We used a flat(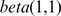) prior for the mean, 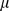, within herd prevalence and a vague (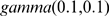) prior for the variance related term 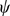.

The prior distributions used for the diagnostic test performances are given in Table 1. The Brucella cELISA has been well studied and data from 6 well described studies was use for the priors [25]. There was very little published data on the Linnodee test, so estimates were made from the manufacturers data sheet supplied with the kit. A number of publications reported using the CHEKIT Q fever kit e.g.. Schelling *et al.* [26], however, none of these reported details of the numbers of animals used to validate the test and we have used relatively vague priors with a mean performance of around 92% and 100% for sensitivity and specificity respectfully.

The model parameters were estimated using a Markov chain Monte Carlo methodology with JAGS software [27] called from R (R core team 2009) using the Rjags package. After an initial burn-in period of 200,000 samples a further 300,000 were collected from 3 McMC chains for posterior inference. Apparent convergence of the McMC samples was assessed by visual examination of the sample histories and calculation of the Brooks-Gelman diagnostic [28].

### Mapping

Herds had been geo-referenced in the initial (2000) study using hand-held GPS device. The spatial distribution of within herd prevalences, 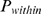, estimated using the Bayesian analysis, were mapped using the R software version 2.9.1 (http://cran.r-project.org/) (Packages ‘Sp’, ‘classInt’, ‘RColorBrewer’ and ‘maptools’). Manual jittering was applied to the plotted location of herds with similar recorded locations in order to separate plotting symbols on the published graphics. Mean estimates of prevalence were mapped to a 7 interval colour scale using the same scale for all three pathogens for comparison purposes.

A provisional exploration of global spatial clustering of seropositive herds was carried out using the Cuzick Edwards' *k*-nearest neighbour test [29]. A herd was classed as positive using a cut-off of 1 for *Brucella* and 2 for the *Leptospira hardjo* and *Coxiella burnetii*. For each seropositive herd the test counts how many *k*-nearest neighbours are also seropositive such that if they are 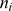 seropositives and 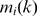 is the number of seropositive herds in the *k* nearest neighbours of her *i* so that 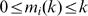, for *i* = 1, …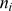, a test statistic 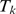 can be calculated as follows:
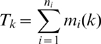
(8)


When seropositives are clustered, the nearest neighbour to a seropositive tends to be another seropositive herd and 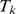 will be large. This is standardised as:
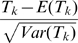
(9)and the p-value reported using the Excel spreadsheet addin by Carpenter (Spatial Statistics - University of California, 1998). One of the main advantages of this non-parametric test statistic is that it takes account of the heterogeneous distribution of the population at risk as positive and negatives are drawn from the same population.

### Herd-Level Sensitivity and Specificity

Using the software tool HERDACC [30] the herd-level sensitivity (HSe) and herd-level specificity (HSp) were explored for a range of true seroprevalences using the point estimates of the test parameters based on the priors in Table 1.

### Ethics Statement

This study used cattle sera biobanked in 2000. The cattle were sampled by a qualified veterinary surgeon with the consent of the animal owner and in accordance with the Cameroonian Ministry of Research (MINREST) guidelines and approval from the University of Liverpool ethics committee in 1999.

## Results

### Descriptive Test Based Results

**Figure 2:**
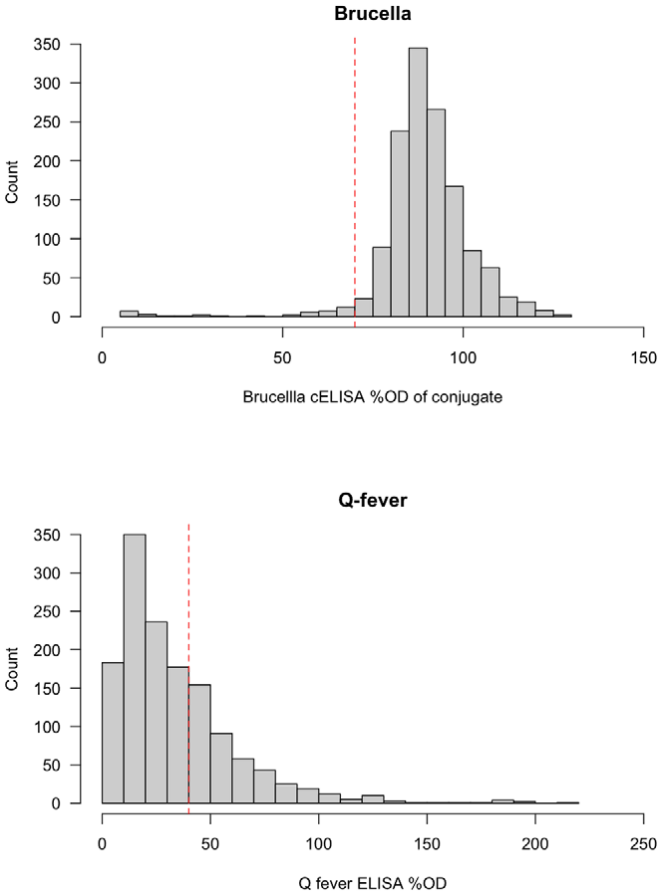
Histogram of optical density values (OD) for the Brucella cELSIA and Q fever ELISA.

A total of 1377 cattle ranging from 8 months to 15 years of age were sampled from 146 herds. The brucella ELISA and Q fever ELISA OD (optical density) values are presented in Figure 2. The distribution of the percentage OD of the conjugate for the Brucella cELISA suggests a large negative population with a small test positive population. The distribution of Q fever OD values does not suggest a clear distinction between the test positive and negative animals at the manufacturers cut-off. The Leptospira ELISA does not produce a continuous OD that is comparable between ELISA test plates.

Table 2 shows the estimates of the region-wide animal-level sero-prevalence (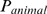), proportion of herds sero-positive (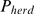) and within-herd animal-level prevalence (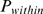). Prevalence estimates are shown both from test data (i.e. apparent prevalence) and from the Bayesian analysis, which adjusts for diagnostic test sensitivity and specificity. The estimated proportion of seropositive herds is shown using two simple rule based approaches. These rules require either one or more, or two or more test positive animals to classify a herd as seropositive.

**Table 2:** Animal-level (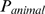), herd-level (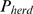) and within herd (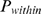) true (model based with 95% highest density intervals) and apparent (with 95% confidence intervals adjusted for study design effects) seroprevalences for cattle in the Adamawa Province of Cameroon to Brucella spp., Leptospira Hardjo and Q fever.

Disease	Parameter	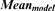	LHDI	UHDI	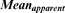	LCI	UCI
Brucellosis	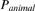				0.031	0.018	0.044
	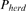	0.203	0.042	0.776	0.159	0.086	0.233
	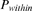	0.161	0.000	0.345	*0.179	0.141	0.218
Leptospirosis	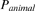				0.304	0.276	0.332
	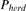	0.945	0.871	1.000	0.933	0.894	0.972
					+0.760	0.685	0.836
	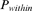	0.357	0.116	0.577	*0.334	0.304	0.364
Q fever	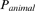				0.313	0.273	0.035
	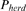	0.681	0.443	1.000	0.853	0.780	0.926
					+0.629	0.519	0.740
	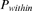	0.393	0.000	0.725	*0.363	0.324	0.403

*The mean for subpop with 1 or more test positives in herd. + Herd-level seroprevalence estimates using a cut-off of 2 test positive animals.

The apparent 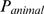 of *Brucella spp.* seropositives was 3.1% whereas *Leptospira hardjo* and Q fever had much higher apparent 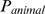 seroprevalences of 30.4% and 31.3% respectively.

About 16% of herds (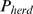) had at least one test positive animal for *Brucella spp.* compared to 93% for *Leptospira hardjo* and 85% for Q fever. In these test positive herds the apparent 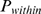 was 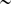
18% for *Brucella spp.* compared to 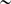
33% for *Leptospira hardjo* and 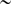
36% for Q fever.


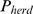
 for each division was estimated for each of the three infection and are given in Table 3. For each infection, approximately similar proportions of herds are sero-positive across the five administrative divisions (*Brucella spp.* Fisher's exact test p = 0.688; *Leptospira hardjo* Fisher's exact test p = 0.526; Q fever Fisher's exact test p = 0.369).

**Table 3:** Herd-level (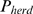) apparent Divisional seroprevalences (with 95% confidence intervals adjusted for study design effects) for cattle in the Adamawa Province of Cameroon to *Brucella spp.*, *Leptospira Hardjo* and Q fever.

Division	Brucella	95% CI	Leptospira	95% CI	Q.fever	95% CI
Vina	0.229	(0.111–0.347)	0.958	(0.901–1.00)	0.875	(0.771–0.979)
			+0.813	(0.691–0.934)	+0.604	(0.440–0.769)
Mbere	0.136	(0.00–0.343)	0.881	(0.757–1.00)	0.763	(0.494–1.00)
			+0.814	(0.683–0.944)	+0.610	(0.284–0.936)
Djerem	0.161	(0.017–0.305)	0.935	(0.850–1.00)	0.774	(0.595–0.954)
			+0.742	(0.483–0.984)	+0.613	(0.368–0.858)
Mayo Banyo	0.091	(0.00–0.274)	0.909	(0.815–1.00)	0.939	(0.858–1.00)
			+0.667	(0.483–0.984)	+0.652	(0.428–0.875)
Faro et Deo	0.133	(0.00–0.298)	1.00		0.933	(0.799–1.00)
			+0.733	(0.483–0.984)	+0.733	(0.353–1.00)

In addition herd-level (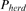) apparent Divisional seroprevalences (+) (with 95% confidence intervals adjusted for study design effects) *Leptospira Hardjo* and Q fever are given after adjusting the herd-level cut-off to be 2 or more test positive animals to class a herd as positive.

### Seroprevalence Results by Age

**Figure 3:**
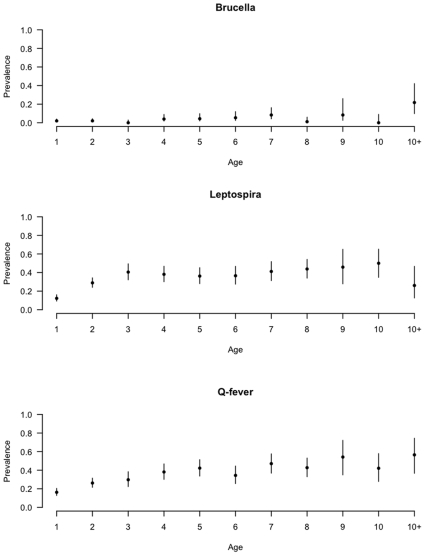
Age stratified animal-level seroprevalence based on raw test results (not adjusted for clustering within herds or diagnostic test imperfections).

The age-stratified apparent seroprevalences for each infection are given in Figure 3. The apparent 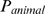 for *Leptospira hardjo* peaks at around 3 years of age and appears to be steady at 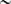
40% of animals thereafter. The pattern for Q fever is a much more gradual rise possibly peaking at around 45–50% by 8 or 9 years of age. In a closed population with a life long immunity and a non zero force of infection across all ages we would expect seroprevalence to increase asymptotically to 1. A lower asymptotic seroprevalence may be due to loss of immunity or introduction of new animals. However, we would anticipate that the numbers entering are limited and that most of the effect will be due to waning immunity. The pattern for Brucellosis is less clear given the very low apparent 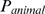 although there is a suggestion of higher seroprevalences in older animals.

### Herd-Level Sensitivity and Specificity

**Figure 4:**
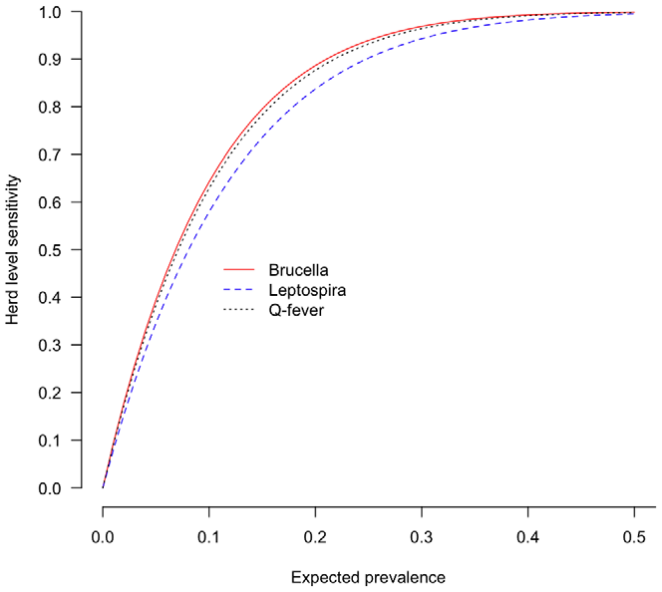
The herd-level sensitivities (HSe) for each of the three infections over a range of true seroprevalences assuming a perfect test specificity.

The original sampling strategy for this survey assumed a 50% within herd prevalence as it was designed to detect foot-and-mouth disease with a 95% herd level sensitivity. Herd-level sensitivity (HSe), which is the probability that a seropositive herd is correctly classified as seropositive, is a function of the sample size, diagnostic test sensitivity, sample interpretation and importantly, within herd animal-level prevalence. The estimated HSe across a range of true within herd seroprevalences are given in Figure 4. Herd-level specificity (HSp) is the probability that a a truly seronegative herd is correctly classified as negative by the test system. However, the HSp is simply a function of the sample size and diagnostic test specificity.

Our results suggest that for *Brucella spp.* the expected prevalence is much lower than the design assumption of 50%. For the *Brucella spp.*, using the literature based estimates of the cELISA test performance, sampling 10 animals per herd and with an expected within herd prevalence of 15% the HSe was estimated to be 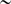
84% and for a seroprevalence of 20% to be 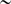
91%. Although high, these results mean that unadjusted estimates of 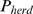 in an area will underestimated.

The HSp decreases as the number of animals sampled increases and is 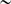
86% for the Brucella cELISA. Therefore, in a completely disease free setting using this testing system we would expect to see on average 21 seropositive herds out of 146. Furthermore, we would expect to only find one false positive animal in a sample of 10 from a herd of 70. Therefore herds with 2, 3 and 4 test positives can more confidently be considered truly seropositive.

The HSe for *Leptospira hardjo* based on the available estimates of diagnostic test performance were 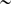
99.2% for an expected 30% true seroprevalence and 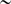
99.8% at 40%. Therefore at the apparent seroprevalences observed the HSe is high. However the herd level specificity (HSp) is very low at 73.3%. Therefore in a truly negative population using this test we would expect to see 39 test positive herds out of 146. However, the HSp can be greatly improved with minimal impact on the HSe by increasing the cut-point from 1 to 2 test positive animals required to be positive to classify the herd as seropositive. This gives an adjusted estimated HSe of 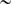
97.7% at 30% and HSp of 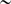
98.1%. This approach was used to re-estimate the overall and Divisional 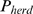 (shown in bold in Tables 2 and 3). This resulted in a new estimated proportion of herds seropositive with *Leptospira hardjo* of 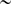
76%, a reduction of 17%.

The HSe for Q fever based on the available estimates of diagnostic test performance were 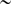
99.6% for an expected seroprevalence of 30% and 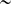
100% for 40%. The HSp was low estimated to be 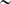
63%. Therefore, in a truly negative population using this test 55 herds would be classified as seropositive out of 146 sampled herds. However, as with the *Leptospira hardjo* test, the HSp can be greatly improved with minimal impact on the HSe by increasing the cut-point from 1 to 2 test positive animals. This gives an adjusted estimated HSe of 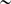
94.8% and HSp of 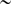
99.3%. The overall and Divisional apparent 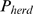 were re-estimated and are given in Tables 2 and 3. This resulted in a new estimated 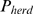 of 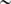
63%, a reduction of 22%.

### Model Based Seroprevalence Estimates Adjusted for Test Performance

Using the hierarchical Bayesian analysis the test imperfections, the uncertainty about their Se and Sp and the study design can all be incorporated to estimate 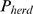 and 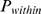. The overall estimates are given in Table 2 for comparison with the apparent seroprevelence estimates. The model's 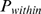 estimates were slightly higher for *Brucella spp.* at 20.3%, similar for *Leptospira hardjo* at 94.5% and lower for Q fever at 68.1% compared to the apparent estimates. These differences reflect the problems of HSe for *Brucella spp.* using the raw test results and the poor HSp of the Q fever ELISA as already discussed.

The hierarchical model allows for a mixture of sero-negative and sero-positive herds and as well as uncertainty in the test parameters. There will be some herds classed as seropositive falsely by having a false test positive animal and there will be herds that are classified as negative due to the sample failing to pick up a seropositive animal. Furthermore the model based approach enables estimation of 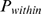 which can not be done in a conventional analysis after shifting the cut-off.

**Figure 5:**
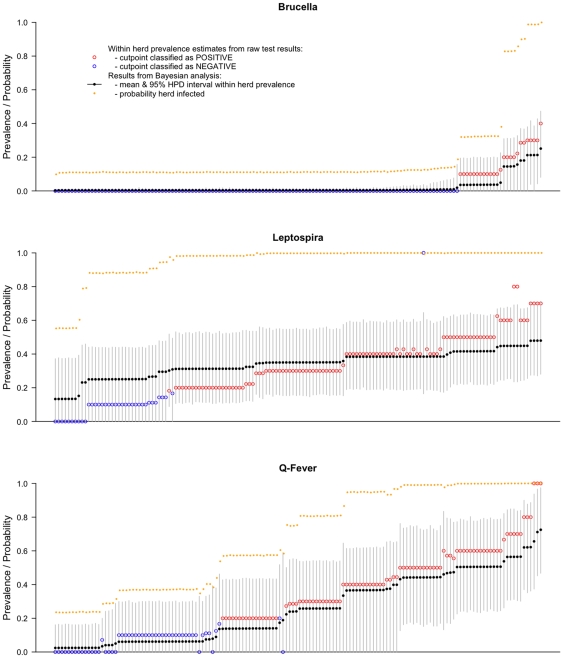
Caterpillar plots showing the classification of each of the 146 herds based on the raw test results and the Bayesian seroprevalence model estimates of true within herd seroprevalence with 95% highest density intervals. Herds are ordered along the x axis based on the estimated within herd seroprevalence.

The model results are summarised for each herd and shown in the caterpillar plots in Figure 5. The posterior mean 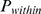 for each herd from the Bayesian analysis is plotted, along with the 95% highest density interval, the apparent seroprevalence from the test results and the probability that the herd was seropositive from the Bayesian analysis.

The graph for *Brucella spp.* still strongly supports the results from the classical analysis and most herds have a low or zero 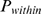 and a low probability test negative herds are seropositive. The model estimates for 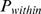 for non zero herds is lower than the estimates from the classical approach consistent with a low positive predictive value for a test positive given the low seroprevalence. The probability that a herd is infected increases once the 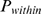 rises above 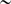
15%.

The graph for *Leptospira hardjo* is more complicated to interpret. The model estimates for each individual 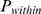 suggest a range of 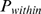 from 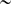
12% to 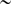
50% compared to the classical estimates that range form 0% to 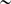
70%. There is a switch-over at 35% seroprevalence from the uncorrected test results underestimating 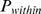 to overestimating it, reflecting the point where Se and Sp switch their influence. As with brucellosis, once the 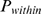 gets above 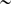
20% the probability that the herd is seropositive increases to above 90% and is 100% when 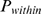 is above 30%. Using the 2 or more test positive animals cut-off appears to largely classify the same herds with near 100% probability from the model. However the herds with 1 test positive (those with 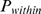 of 10%) have a very high probability of being seropositive from the model. There is one herd that due to the small sample of only one animal had a 100% test seroprevalence but the model predicted a more modest 40% true seroprevalence.

The figure for Q fever firstly shows the higher uncertainty in the estimates due to the lack of precision in the Se and Sp estimates. There also appears to be a much wider range of 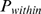 from 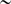
5% to 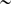
70%. The use of the higher cut-point reclassifies many of the lower prevalence herds as seronegative; in the model they have a low probability of being seropositive until 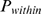 gets above 30% when the probability the herd is seropositive gets above 95%. This reflects the lack of certainty in the test parameters compounded by the small sample from each herd.

### Distribution of Seropositive Herds

**Figure 6:**
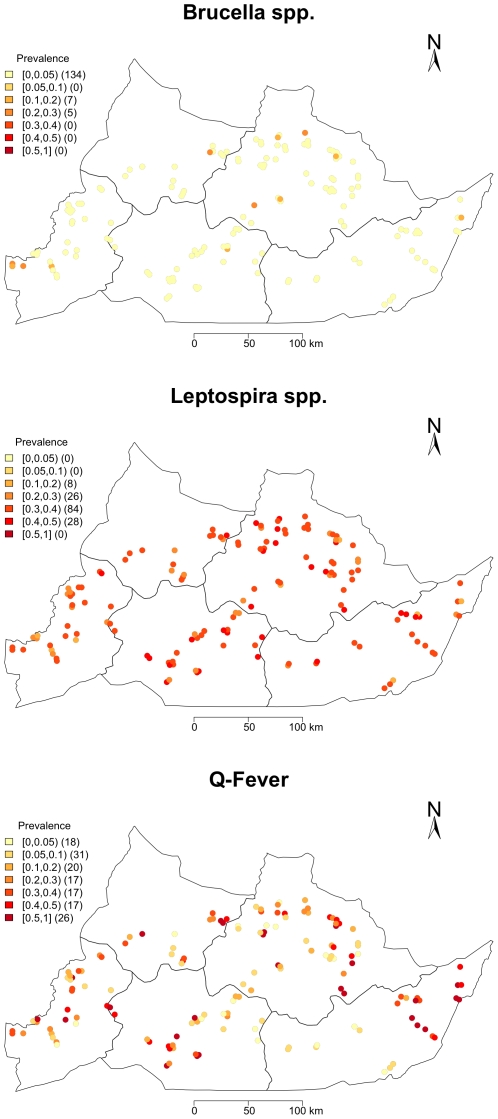
Spatial distribution of sampled herds in the Adamawa Region of Cameroon showing estimated within herd seroprevalence for brucellosis, leptospirosis and Q fever.

The spatial distribution of the 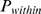 estimates from the model are plotted in Figure 6. The results of the Cuzick Edwards test statistic are given in Table 4. The spatial distribution of *Brucella spp.* seropositive herds is thinly dispersed across the Region with some suggestion of clustering in the west which is supported by the highly significant test statistic (p
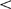
0.001) at all levels up to the third nearest neighbour. In contrast the spatial distribution for *Leptospira hardjo* 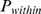 estimates suggest high seroprevalence herds across the entire Region and little statistic evidence of clustering. The pattern for Q fever is the most interesting with a much more variation in 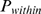 distribution across the Divisions and possible clustering around the major Divisional towns which was supported by the Cuzick Edwards test statistic (p
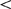
0.001) at all levels up to the third nearest neighbour.

**Table 4:** Cuzick Edwards 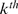 nearest neighbour analysis results for brucellosis, leptospirosis and Q fever in 146 randomly sampled herds from the Adamawa Region of Cameroon.

k	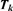	E( 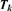 )	V( 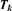 )	p-value
*Brucella spp.*				
1	11	3.8	4.9	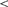 0.001
2	17	7.6	10.0	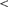 0.001
3	24	11.4	15.2	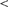 0.001
*Leptospira hardjo*				
1	81	84.2	16.4	0.786
2	167	168.4	33.0	0.597
3	245	252.6	63.0	0.832
Q fever				
1	81	59.0	20.6	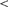 0.001
2	147	448.0	41.8	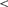 0.001
3	204	177.0	74.2	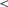 0.001

## Discussion

This serological analysis of exposure to *Brucella spp.*, *Leptospira spp.* and *Coxiella burnetti* in cattle is the first report from a well described population based sample of herds in the Adamawa Region of Cameroon for several decades. We have estimated the seroprevalences of these three diseases using both a classical approach which allows for some adjustment for the multi-level design and a model based approach that allows incorporation of the multi-level design of the original sampling, the sensitivity and specificity of the diagnostic tests used and the uncertainties in these tests. The caterpillar plots in Figure 5 summarize most of the information in the results and show that particularly for leptospirosis and Q fever there is a large uncertainty in the individual within herd estimates due to the small sample size from each herd of only 10 animals. The model approach also has the advantage that herds where only a few animals were sampled are adjusted for the general seroprevalence avoiding overestimation. However, for these two diseases it also does confirm the high level of probability that these herds have been exposed. It also highlights the need for high quality diagnostic tests with well described characteristics in order to make reliable interpretation of serological surveys. The lack of sensitivity and/or specificity need to be adjusted for in order to get unbiased estimates of seroprevalence and as we have shown here that failure to do so can give significantly different estimates.

These analyses estimate the seroprevalence of brucellosis to be much lower than expected even after adjustment for the design and diagnostic test performance. The reasons are not clear. Seropositive herds appear to be focused mainly around the Regional capitol, Ngaoundere, and the western border area next to the North Western Region and Nigeria. The study was under powered to detect seropositive herds at these low within herd seroprevalences and this is therefore likely to be an underestimate of the problem. However, the animal-level seroprevalence is robust.

It is estimated that around 61% of the known 1415 human pathogens are zoonotic [2]. The concept of ‘one medicine’ which is defined as the science of all human and animal health diseases has been around for several decades but its uptake is still generally is lacking in many developing countries where it could have most impact [31]. Interestingly Cameroon has a very extensive veterinary infrastructure with 88 centres in the Adamawa alone. Understanding the epidemiology of diseases such as brucellosis, leptospirosis and Q fever are important veterinary issues relating to production losses and abortions. However, the zoonotic nature of these diseases means that it is also important for the medical profession to understand the extent and prevalence of these diseases in the livestock reservoir. All three diseases produce very variable non-specific symptoms in people and are generally believed to be hugely under reported largely due to confusion with malaria in developing countries where 50–80% of malaria cases may suffer fevers resulting from other causes [32].

*Brucella* seroprevalence in the cattle population of the Adamawa Region appears to be very low with only around 3% of animals in 20% of herds and a mean within herd seroprevalence of 16%. Reports from the literature suggest a very variable brucellosis seroprevalence at individual and herd-level across study regions. Estimates include animal-level seroprevalences of 20.2% in Sudan [7], between 0.3% and 8.2% in Eritrea [33], 12.3% in Tanzania [34], 6.6% in Chad [26], 3.3% in the Central African Republic [35], 14.1% to 28.1% in Zambia [36]. At the herd/unit level estimates range from 2.4% and 46.1% under different husbandry systems in Eritrea [33] and in Zambia from 46.2% to 74% across study areas [36]. Despite the lack of official reports on brucellosis in Cameroon since 1996 (OIE, handistatus II, http://www.oie.int/hs2/), the disease is believed to still be endemic across the country [37] and the same authors working in Western Province estimated seroprevalaence to be 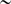
10% in cattle sampled at an abattoir. A number of studies have been carried out, mainly in the Northern Province, where seroprevalence values ranging form 7.5% to 31% have been reported [38]–[40], although these estimates may be largely affected by the sampling method and diagnostic techniques. The low seroprevalence and apparent decline since the 1980s may be due to improved husbandry and awareness but we currently have no knowledge of any systematic control efforts or education campaigns having been carried out.

There does not appear to be any reliable up-to-date information on human brucellosis for the region [41]. However, the sub-Saharan African countries included by Pappas (et al.) [41] appear to have lower annual incidence than North African countries. This may however reflect a poor reporting system in many sub-Saharan regions. There is considerable data on risk factors for human brucellosis and drinking unpasteurized milk [42] and handling abortive materials [43] from livestock as well as professions such as herdsman and abattoir worker [44] are all higher risk. Currently there are no programs aimed at controlling or eradicating brucellosis from the region. New penside/home test tools are now available for the testing of animals [25], [45] and humans [46] that could greatly speed up identification and of infected animals and people and make control a real possibility.

There are only a few published reports on leptospiosis in African livestock and human populations. Serological studies in cattle in various African countries report overall leptospiral serovars prevalences of 10.4% [47] to 27% [48] in Zimbabwe, of 21% [49] in Malawai and 45% [50] in Mali. There is also one report of a seroprevalence of 22% in pigs in South Africa [51]. No livestock cases have been reported in Cameroon in the last 10 years (OIE, handistatus II, http://www.oie.int/hs2/). Serological surveillance of human patients in Africa show a similar high seroprevalence with reports from Senegal of a seroprevalence of 35% [52] in hospital patients compared to 37% to 64% in different patient groups in Somalia [53] and 15.7% in gold miners in Gabon [54].

Q fever has been recently reviewed [14] but cites only one paper for Africa [14]. Malawian zebu cattle have shown seroprevalence ranging from 1.5% up to 5% [55]; 7%–8.5% for cattle in Transvaal [56]; 39% for cattle in Zimbabwe [57]; 4% in Chad [26]. No livestock cases have been reported in Cameroon in the last 10 years (OIE, handistatus II, http://www.oie.int/hs2/). The seroprevalence in 5 herds in Zambia were 0.9% [58]. In human populations estimates for the general population are lacking. In a hospital based study in Mali [59] 40% of patients admitted with fever where positive but none of the individuals had been diagnosed with Q fever at their initial examination.

The Bayesian modeling approach proved useful as this allowed the incorporation of the diagnostic test Se and Sp, the uncertainties in these parameters and the study design features. One of the clear implications of this estimation process is that the within herd sample sizes were small in terms of estimating within herd seroprevalences, which they were never intended for in the first place. However, this approach has allowed unbiased estimates of seroprevelance from a design that was not intended for studying these diseases, allowing maximum information to be extracted from such a survey and the banked material and providing robust estimates for these infections. This is not possible from a classical statistical analysis.

This study points to the need for further investigations of these diseases in the Region to confirm the initial and findings and to estimate the levels of clinical and sub-clinical disease in both the livestock and human populations in order to prioritize control strategies. However, control of these diseases in the livestock may be difficult in extensive pastoralist communities in SSA and will need to include education on handling and disposal of abortive materials.

The seroprevalence of brucellosis, leptospirosis and Q fever were estimated for the Adamawa Region of Cameroon and brucellosis was found to have a low seroprevalence at both the animal and herd-level compared to leptospirosis and Q fever. The low brucellosis seroprevalence was unexpected based on previous studies from the literature. The high seroprevaelences of exposure to *Leptospira spp.* and *Coxiella burnetti* represent a major challenge both from a veterinary and a public health view point. It is likely that there is a high incidence of abortion/reproductive failure in affected herds leading to potentially high levels of exposure of livestock owners and their families which is then not being correctly diagnosed. Further studies are clearly needed to study these important zoonoses and to be able to understand the human and animal interactions and the clinical significance of these seroprevalences in both the animal and for the human populations.
